# The Role of Circulating Tumor Cells in the Prognosis of Local Recurrence and Local Residual Nasopharyngeal Carcinoma Undergoing Endoscopic Resection

**DOI:** 10.1155/2022/1453792

**Published:** 2022-09-12

**Authors:** Liang Tang, Zhifeng Guan, Mengdi Xu

**Affiliations:** ^1^Department of Otorhinolaryngology, Minzu Hospital of Guangxi Zhuang Autonomous Region, Guangxi, China; ^2^Key Laboratory of Early Prevention and Treatment for Regional High Frequency Tumor (Guangxi Medical University), Ministry of Education, Guangxi, China; ^3^Guangxi Key Laboratory of Early Prevention and Treatment for Regional High Frequency Tumor, Guangxi, China; ^4^Department of Radiotherapy, Tumor Hospital Affiliated to Nantong University, Nantong Tumor Hospital, Nantong 226000, China; ^5^Zhejiang Baiyining Medical Laboratory Co,.Ltd, Zhejiang, China

## Abstract

**Purpose:**

To investigate the role of circulating tumor cells in the prognosis of local recurrence and local residual nasopharyngeal carcinoma undergoing endoscopic surgery.

**Methods:**

A total of 56 patients with locally residual nasopharyngeal carcinoma (NPC) who underwent nasal endoscopic surgery from August 2018 to December 2021 were included. The status of circulating tumor cells (CTC) before and after surgery was detected, and its relationship with clinical characteristics and postoperative survival was analyzed.

**Results:**

After nasal endoscopy, the positive rates of CTC and mesenchymal CTC (MCTC) detected in patients with nasopharyngeal carcinoma were significantly lower than those before treatment (*P*=0.0376; *P*=0.0212). Before nasal endoscopy, the status of CTC and MCTC was significantly correlated with the *T* stage (*P* < 0.05). After nasal endoscopy, the status of CTC and MCTC was significantly correlated with the TNM stage, *T* stage, and first radiotherapy mode (*P* < 0.05). The PFS of patients with different clinical characteristics was analyzed, and the results showed that the PFS of NPC patients with CTC (+) was significantly shorter than that of CTC (−) patients (18.71 vs. 22.47, *P* < 0.05) and the PFS of NPC patients with MCTC (+) was significantly shorter than that of MCTC (−) patients (18.22 vs. 22.30, *P* < 0.05). The PFS of NPC patients in TNM stage (I-II) was significantly longer than that in TNM stage (III) patients (22.53 vs. 18.57, *P* < 0.05). The PFS of NPC patients whose first radiotherapy mode was conventional was significantly longer than that of patients whose first radiotherapy mode was enhanced (22.14 vs. 16.85, *P* < 0.05). The COX analysis showed that MCTC and TNM stages were independent risk factors affecting the prognosis of local recurrence or local residual nasopharyngeal carcinoma after endoscopic resection (*P* < 0.05).

**Conclusion:**

The detection of CTC is helpful for the prognosis evaluation of local recurrence or local residual NPC after endoscopic resection of NPC. The MCTC is an important factor affecting the prognosis of NPC patients.

## 1. Introduction

Nasopharyngeal carcinoma (NPC) is one of the most common malignant tumors in China. The current treatment for NPC is radiotherapy, with or without chemotherapy. This treatment mode has achieved ideal efficacy [1]. However, there are cases with local residual tumor cells after radiotherapy. A recent study reported that 14% of 3328 NPC patients who received radiotherapy as the primary treatment modality had local residual or local recurrence [2]. In recent years, with the deepening research on endoscopic skull base anatomy, the increasingly sophisticated endoscopic equipment and instruments, and the sophisticated surgical techniques, transnasal endoscopic resection has become an important alternative option for patients with locally residual or locally recurring NPC [3]. However, transnasal endoscopic is a local treatment, and a comprehensive treatment (radiotherapy or chemotherapy) using various methods is still required after surgery according to different histological types and clinical stages to achieve the best curative effect. Therefore, the prognostic evaluation of transnasal endoscopic resection is very important for clinical decisions.

Circulating tumor cells (CTCs) are the general term for various types of tumor cells shed from solid tumors into the peripheral circulation [4]. It is believed that CD45−/EpCAM+/CK+ cells in peripheral blood are CTCs, and their status is closely related to the clinical stage, lymph node metastasis, degree of differentiation, and prognosis of tumor patients and plays an important role in tumor recurrence, metastasis, and prognosis evaluation [5]. Some studies have indicated that the progression-free survival and overall survival of cancer patients with CTCs in peripheral blood are significantly shortened [6]. At present, CTC has been used in the prognostic evaluation of various tumors [7, 8], but it is rarely used to evaluate the prognosis of patients with locally residual or locally recurrent NPC in patients undergoing endoscopic resection. This study evaluated the role of CTC in the prognosis of NPC.

## 2. Materials and Methods

### 2.1. Research Subject

A total of 56 patients with local recurrence and local residual NPC who underwent nasal endoscopic treatment in Nantong Tumor Hospital from August 2018 to December 2021 were included. Inclusion criteria were as follows: (1) Patients with locally recurrent NPC, which is defined by local tumor recurrence observed by nasal endoscopy after 6 months of radiotherapy. Tissues were collected and confirmed by pathological examination. (2) Patients with local residual disease, which was defined by a residual mass in the nasopharynx and/or parapharyngeal space, were found by nasal endoscopy and MRI after 3 months of radiotherapy. Tumor tissue was collected and confirmed by pathological examination. (3) Patients were treated with radiotherapy for nasopharyngeal carcinoma. Reradiotherapy in the short term may cause serious complications such as bone necrosis, radiation-induced brain cell damage, internal carotid artery rupture, and hemorrhage, and they cannot continue to receive radiotherapy. (4) The patient's general condition is good and can tolerate general anesthesia surgery. Exclusion criteria were as follows: (1) Patients with intracranial metastatic lesions revealed by CT or MRI examinations of the cranial brain, nasopharynx, and paranasal sinuses. (2) Patients with distant metastases. (3) Patients with surgical contraindications, such as patients with severe liver, kidney, cardiac insufficiency, and severe coagulation dysfunction.

## 3. Detection of CTCs in Peripheral Blood

### 3.1. Enrichment of CTCs

After collecting 15 ml of peripheral venous blood from patients with local recurrence or local residual NPC in an anticoagulation tube, it was shaken gently to prevent blood coagulation. Blood samples were collected one week before surgery and one month after surgery. The sample was centrifuged (2500 r/min 5 min) to remove the supernatant and was fixed for 8 min. After centrifugation, the pellet was mixed in 100 ml of 10*∗*PBS for 10 seconds. . Then, 900 ml of water was added and centrifuged at 1000 rpm for 5 min until no red blood cells are seen in the pellet. The filter was connected to a vacuum pump through a vacuum manifold, and the liquid in the sample was transferred to the filter and filtrated through an 8 *μ*m pore size filter. CTCs and some leukocytes are trapped in the filter. The filtration membrane was fixed with formaldehyde solution at room temperature for 60 min.

### 3.2. Incubation and Staining

The samples were incubated with a permeabilizer for 5 min and washed three times with PBS solution 0.1 × SSC (Sigma, St. Louis, USA). The samples were incubated with digestive enzymes for 60 min and washed three times with PBS. After adding the probe working solution (specific capture probes: epithelial biomarker probes EpCAM, CK8/18/19, mesenchymal biomarker probes vimentin and twist, and leukocyte marker CD45), the sample was placed in a 40°C biochemical incubator for 3 h and washed three times with PBS. Then, the preamplification working solution (30% horse serum, 1.5% sodium dodecyl sulfate, 3 mMI Tris-HCl (pH 8.0), and 0.5 fmol preamplification probe) was added to the sample and incubated for 30 min in a 40°C biochemical incubator. The sample was washed with PBS three times before adding amplification working solution (30% horse serum, 1.5% sodium dodecyl sulfate, 3 mMI Tris-HCl (pH 8.0), and 1 fmol amplification probe) and placed in a 40°C biochemical incubator for 30 min and again washed three times with PBS.

We used a fluorophore-labeled probe to hybridize with the amplification probe to generate a fluorescent signal. Add the fluorescent dyes Alexa Fluor 488 (interstitial biomarker probe vimentin and twist) and Alexa Fluor 750 (for labeling leukocytes) (Marker CD45) to the sample and incubate for 30 min in a 40°C biochemical incubator. The sample was washed three times with PBS before Antiquencher (containing DAPI for nuclear fluorescent staining) was added to the sample. The results were observed directly after the samples were placed for 5 min.

### 3.3. Interpretation of Results

After hybridization and staining with different specific probes, different fluorescent signals can be identified. Interstitial CTC (MCTC) is a type of CTC. This study mainly explored the status of CTC and MCTC. The peripheral blood CTC≥ 3/5 ml of patients with nasopharyngeal carcinoma was judged as positive, and <3/5 ml was judged as CTC negative. MCTC≥ 1/5 ml was judged as positive, and <1/5 ml was judged as CTC negative.

### 3.4. Surgical Procedure

We kept the patient in a supine position, inserted a special trachea into the patient's trachea through the mouth, and performed general anesthesia. Then, we used cotton piece to take 0.1% of adrenalin to make nasal mucosa of the patient fully contracted. The mucosa 5 mm away from the residual lesion is cut under the nasal endoscope using a high-frequency electric knife or a low-temperature plasma knife until they reach the bone. The mucosa and the bone surface are separated with a nerve dissection stick close to the incision, thereby excising the lesions. After the operation, the nostrils were stuffed and washed using a large amount of distilled water to completely clean the cavity and compress the bleeding. When special circumstances are encountered during the operation, such as the soft tissue of the nasopharynx cannot be cut at one time, the method of partial excision is adopted. Meanwhile, the tissue 1 cm behind the turbinate around the malignant tumor was treated with hemostasis. Postoperative adjuvant chemotherapy was given to the patient. Adjuvant chemotherapy was a TP regimen: docetaxel 75 mg/m^2^, d1. Cisplatin25 mg/m^2^, d1–3. One cycle lasted for 21 days, and the patient received chemotherapy for 2-3 consecutive cycles.

### 3.5. Postoperative Follow-Up

All patients were followed up by telephone after endoscopic nasal resection. Follow-up was terminated when the patient's disease progressed, died, or did not respond at the last follow-up. The end of follow-up in this study was January 1, 2022. The median follow-up time was 20 months. PFS is the time from the first day after surgery to the patient's first disease progression or death from any cause. The interval between recurrences was the time from the end of radiotherapy to the time when recurrences were confirmed by examination.

## 4. Results

### 4.1. Changes in CTC Status before and after Treatment

**Figure 1:**
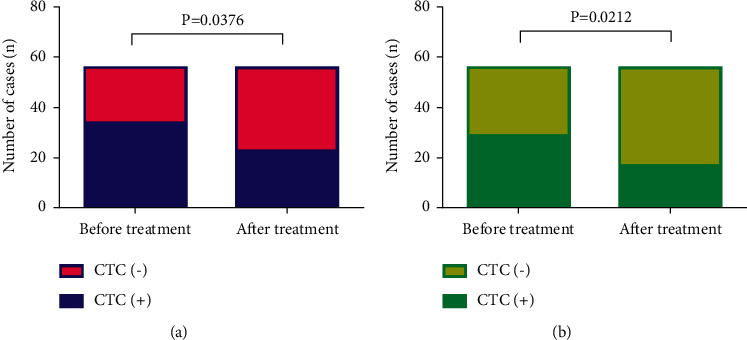
Changes in CTC expression before and after treatment.

Before undergoing transnasal endoscopic resection, 34 out of 56 patients (60.71%) had positive CTCs with a median count of 6.00 CTCs (range, 0–58 per 5 mL of blood). 29 (51.79%) patients were positive for MCTCs with a median count of 5.00 CTCs (range, 0–64 per 5 mL of blood). At 1 month after transnasal endoscopy, CTCs were detected in 23 (41.07%) patients with a median count of 5.00 CTCs (range, 0–48 per 5 mL of blood); and 17 (51.79%) patients were positive for MCTCs, with a median count of 3.00 CTCs (range, 0–39 per 5 mL of blood). After nasal endoscopy, the positive rates of CTC and MCTC detected in patients with nasopharyngeal carcinoma were significantly lower than those before treatment (*c*2 = 4.323, *P*=0.0376; *c*2 = 5.312, *P*=0.0212) (Figure 1).

### 4.2. CTC Status and Clinical Features

Before nasal endoscopy, the status of CTC and MCTC was significantly correlated with *T* stage (*P* < 0.05), but had no significant correlation with age, gender, TNM stage, *N* stage, *M* stage, tumor state, and the first course of radiotherapy (*P* > 0.05). After nasal endoscopy, the status of CTC and MCTC was significantly correlated with TNM staging, *T* staging, and the first course of radiotherapy (*P* < 0.05), but not with age, gender, *N* staging, *M* staging, and the tumor state (*P* > 0.05) (Tables 1 and 2).

### 4.3. PFS Analysis

**Figure 2:**
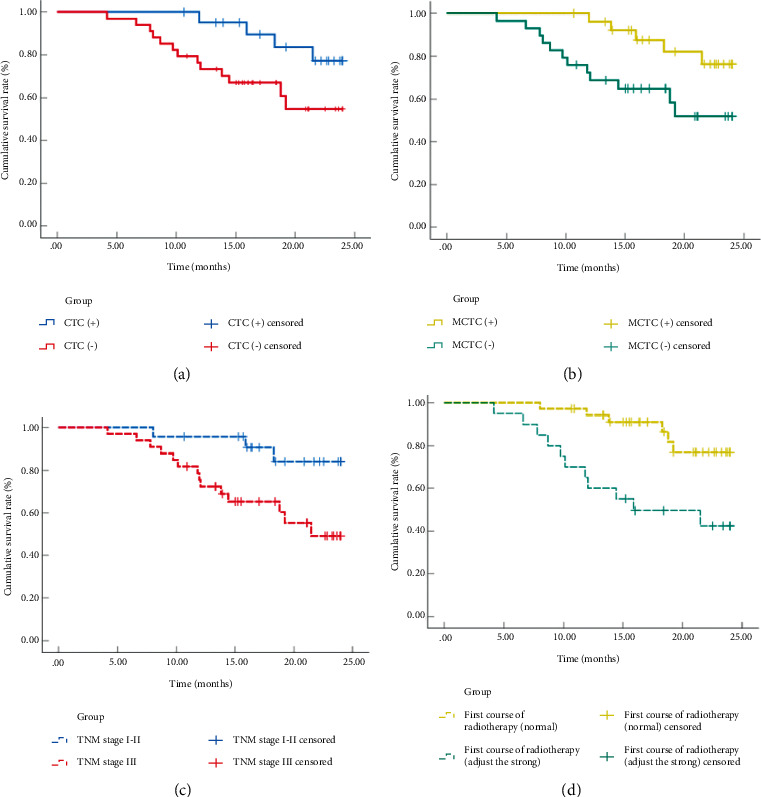
PFS analysis.

During the follow-up period, no patients died. The disease progressed in 16 patients. Five patients had a second recurrence in the nasopharynx. Six patients had a first recurrence in the nasopharynx. Primary second tumors developed in 5 patients, 3 of the tongue and 2 of the palate. The PFS of patients with different clinical characteristics was analyzed, and the results showed that the PFS of NPC patients with CTC (+) was significantly shorter than that of CTC (−) patients (18.71 vs. 22.47, *P* < 0.05). The PFS of NPC patients with MCTC (+) was significantly shorter than that of MCTC (−) patients (18.22 vs. 22.30, *P* < 0.05). The PFS of NPC patients in TNM stage (I-II) was significantly longer than that in TNM stage (III) patients (22.53 vs. 18.57, *P* < 0.05). The PFS of NPC patients was significantly longer in patients whose first radiotherapy mode was conventional than in patients whose first radiotherapy mode was enhanced (22.14 vs. 16.85, *P* < 0.05). There was no significant difference in PFS between the CTC (+) group, the CTC (−) group, the MCTC (+) group, and the MCTC (−) group *D* (*P* > 0.05) (Tables 3 and 4, Figure 2).

### 4.4. COX Univariate and Multivariate Analyses

According to COX analysis, MCTC and TNM staging were independent risk factors affecting the prognosis of local recurrence or local residual nasopharyngeal carcinoma after endoscopic resection (*P* < 0.05) (Tables 5 and 6).

## 5. Discussion

For NPC patients with residual or locally recurrent lesions, given the high incidence of severe toxicity associated with reirradiation, surgery should be considered for locally recurring and resectable cases. Transnasal endoscopic surgical resection is often used clinically. Compared with traditional surgical methods, transnasal endoscopic resection results in less trauma, less damage to surrounding tissues, and less bleeding. In addition, transnasal endoscopic resection has a clearer vision, a thin mirror body, and lesions can be observed from multiple angles, which improves the complete removal of the tumor [9]. After resection, patients with NPC still need to receive reradiotherapy or adjuvant chemotherapy to consolidate the curative effect. Therefore, a reasonable evaluation of the postoperative prognosis of NPC patients is crucial for determining the follow-up treatment [10]. TNM staging is considered to be the most valuable prognostic factor affecting the treatment of nasopharyngeal carcinoma. However, TNM staging only considers the anatomical location of the tumor and does not take into account the heterogeneity of the tumor. Thus, in clinical practice, the same treatment might lead to a completely different effect on patients with the same TNM staging. Therefore, it is of great value to screen out economic, objective, and easily detectable indicators to supplement TNM staging to predict the prognosis of patients with nasopharyngeal carcinoma.

CTC detection has become the representative of the emerging liquid biopsy nowadays. Compared with traditional histopathological biopsy, CTC detection has the advantages of convenience, noninvasiveness, and real-time results and has been widely used in the diagnosis of various tumor diseases and the evaluation of patient prognosis.

CTCs are derived from clones at the primary tumor site and have various cellular phenotypes, including epithelial CTCs, mixed epithelial and mesenchymal CTCs, and mesenchymal CTCs (MCTCs). Cell phenotype can reflect the malignancy of tumor cells, among which MCTC has stronger migration and invasion ability than other phenotypes of CTC, which is also closely related to the clinical characteristics of tumor patients. Si et al. [11] reported that CTC levels were closely related to NPC staging, and total CTC counts were significantly positively correlated with NPC clinical staging. The results of this study also showed that the status of CTC and MCTC was significantly correlated with *T* staging both before and after surgery (*P* < 0.05). In addition, the status of CTC and MCTC after operation was also significantly correlated with TNM staging and the first radiotherapy pattern, indicating that CTC detection is of great significance for NPC patients regardless of before and after operation. However, there is some controversy about the correlation between CTC and TNM staging. The studies of Li et al. [12, 13] clearly pointed out that the positive rate of CTC in NPC patients has no significant correlation with TNM staging. Therefore, expanding the sample size, revalidating the correlation of CTC status with TNM is warranted in future experiments.

CTCs are also related to the prognosis of tumor patients. Studies have reported that CTC-positive breast cancer, colorectal cancer, and liver cancer have a poor prognosis and shorter survival time [14–17]. In nasopharyngeal carcinoma, Zhang [18] found that after 2–4 cycles of chemotherapy, the CTC count of NPC was reduced remarkably, and the change in CTC number was correlated with treatment response. Qian [19] reported that the reduction of CTC was related to the treatment effect of patients with advanced NPC, and the positive CTC before treatment was an independent risk factor for poor prognosis. Yu [20] reported that CTC was associated with poor survival in NPC patients, which may be an independent prognostic factor affecting NPC. In our study, the positive rates of CTC and MCTC detected in patients with nasopharyngeal carcinoma after nasal endoscopy were significantly lower than those before treatment, suggesting that CTC and MCTC counts changed before and after endoscopic nasal resection. For transnasal endoscopic resection, CTC and MCTC are also potential indicators to monitor the prognosis of NPC patients. The PFS of patients with different clinical characteristics was analyzed, and the results showed that the PFS of CTC-positive NPC patients was significantly shorter than that of CTC-negative patients, and the PFS of MCTC-positive NPC patients was significantly shorter than that of MCTC-negative patients. The PFS of NPC patients in TNM stage (I-II) was significantly longer than that of TNM stage (III) patients, and the PFS of NPC patients whose first radiotherapy mode was conventional was significantly longer than that of patients whose first radiotherapy mode was enhanced. In general, the efficacy of intensive radiotherapy is better. However, in this study, the prognosis of early conventional radiotherapy was better than that of intensive radiotherapy. After analysis, we believe that there are two reasons. The first reason is that the radiation dose received by the nasopharyngeal tissue in patients with early intensive radiotherapy is close to the tolerance limit of the surrounding normal structures. When the lesion recurs, the structure of the nasopharynx may change significantly, which affects the prognosis to a certain extent. In addition, there are many important tissues and organs around the nasopharynx. Intensive radiotherapy will aggravate the damage to the surrounding tissues and will also have a certain impact on the prognosis. Another reason is the small number of cases in this study, the short follow-up time, and the lack of analysis of the long-term adverse reactions of the patients. Therefore, whether the first radiotherapy method affects the prognosis of NPC recurrence needs to be further explored. The COX analysis screened out that MCTC and TNM staging were independent risk factors affecting the prognosis of local recurrence or local residual NPC after endoscopic resection, suggesting that MCTC is an important factor affecting the prognosis of NPC patients.

In conclusion, the detection of CTC is helpful for the prognosis evaluation after endoscopic nasal resection of local recurrence or local residual NPC. MCTC is an important factor affecting the prognosis of patients with NPC.

## Figures and Tables

**Table 1:** Correlation between preoperative CTC status and clinical characteristics.

Items	CTC		*P*	MCTC		*P*
	*N* (*n* = 22)	*P* (*n* = 34)		*N* (*n* = 27)	*P* (*n* = 29)	
Age						
≤60	12	20	0.7520	14	18	0.4401
>60	10	14		13	11	

Gender						
Male	14	23	0.7569	17	20	0.6355
Female	8	11		10	9	

TNM stage						
I-II	12	18	0.9064	15	11	0.1864
III	10	16		12	18	

T stage (infiltration depth)						
T1-T2	15	12	**0.0162**	20	7	**0.0002**
T3-T4	7	22		7	22	

N stage (lymphatic metastasis)						
N0-1	12	21	0.5917	17	16	0.5538
N2	10	13		10	13	

Tumor state						
Local recurrence	12	21	0.8876	15	18	0.6206
Local remnants	10	13		12	11	

First course of radiotherapy						
Normal	17	19	0.1028	20	16	0.1402
Adjust the strong	5	15		7	13	

Time between recurrence (years)						
≤2	4	8	0.7844	5	7	0.7411
>2	8	13		10	11	

Note: *N* means negative. *P* means positive.

**Table 2:** Correlation between postoperative CTC status and clinical characteristics.

Items	CTC		*P*	MCTC		*P*
	*N* (*n* = 33)	*P* (*n* = 23)		*N* (*n* = 39)	*P* (*n* = 17)	
Age						
≤60	16	16	0.1168	21	11	0.4502
>60	17	7		18	6	

Gender						
Male	23	14	0.4925	26	11	0.8867
Female	10	9		13	6	

TNM stage						
I-II	20	3	**0.0004**	22	1	**0.0004**
III	13	20		17	16	

*T* stage (infiltration depth)						
T1-T2	20	7	**0.0262**	23	4	**0.0147**
T3-T4	13	16		16	13	

*N* stage (lymphatic metastasis)						
N0-1	19	14	0.8053	23	10	0.9916
N2	14	9		16	7	

Tumor state						
Local recurrence	18	15	0.4245	21	12	0.2416
Local remnants	15	8		18	5	

First course of radiotherapy						
Normal	25	11	**0.0319**	29	7	**0.0172**
Adjust the strong	8	12		10	10	

Time between recurrence (years)						
≤2	6	4	0.6782	7	3	0.6163
>2	12	11		14	9	

Note: *N* means negative. *P* means positive.

**Table 3:** PFS analysis.

Subject	PFS	95% CI	*P*
CTC			
(+)	18.71 ± 1.18	16.39–21.04	**0.0481**
(−)	22.47 ± 0.75	21.01–23.93	

MCTC			
(+)	18.22 ± 1.31	15.64–20.81	**0.0027**
(−)	22.30 ± 0.72	20.88–23.71	

Age			
≤60	19.10 ± 1.15	16.86–21.34	0.0782
>60	21.81 ± 1.01	19.82–23.79	

Gender			
Male	20.19 ± 1.01	18.22–22.16	0.9345
Female	20.23 ± 1.37	17.54–22.92	

TNM stage			
I-II	22.53 ± 0.83	20.91–24.15	**0.0164**
III	18.57 ± 1.16	16.28–20.86	

*T*			
T1-T2	20.99 ± 1.01	19.02–22.97	0.3587
T3	19.41 ± 1.23	17.00–21.83	

*N*			
N0-1	21.07 ± 0.94	19.23–22.91	0.2088
N2	18.98 ± 1.41	16.22–21.74	

Tumor state			
Local recurrence	20.86 ± 0.98	18.96–22.78	0.3352
Local residual	19.34 ± 1.36	16.68–21.99	

First course of radiotherapy			
Normal	22.14 ± 0.71	20.76–23.52	**0.0026**
Adjust the strong	16.85 ± 1.61	13.69–20.00	

Time between recurrence (years)			
>2	21.93 ± 1.35	19.29–24.58	0.4460
≤2	20.31 ± 1.30	17.75–22.86	

**Table 4:** PFS analysis of different CTCS and MCTC status.

Subject	PFS	95% CI	*P*
CTC (+)			
Local recurrence	19.56 ± 1.38	16.86–22.25	0.4841
Local residual	17.17 ± 2.07	13.12–21.22	

CTC (−)			
Local recurrence	22.91 ± 1.04	20.86–24.96	0.2257
Local residual	21.91 ± 1.06	19.83–23.97	

MCTC (+)			
Local recurrence	19.51 ± 1.51	16.55–22.47	0.2776
Local residual	16.03 ± 2.27	11.59–20.47	

MCTC (−)			
Local recurrence	22.41 ± 1.04	20.37–24.46	0.5587
Local residual	22.21 ± 0.92	20.42–24.01	

**Table 5:** COX univariate analysis.

Items	*B*	SE	Wald	Df	*P*	Exp (B)	95.00% CI	
							Upper	Lower
CTC	1.091	0.5782	3.566	1	0.0594	2.978	0.9608	9.241
MCTC	1.126	0.5363	4.408	1	**0.0361**	3.083	1.078	8.818
Age	−0.969	0.5726	2.871	1	0.0921	0.3791	0.1245	1.164
Gender	−0.042	0.5094	0.0071	1	0.9342	0.9597	0.3538	2.601
TNM stage	1.418	0.6383	4.947	1	**0.0263**	4.130	1.183	14.41
*T*	0.4501	0.4942	0.8293	1	0.3622	1.568	0.5952	4.130
*N*	0.6042	0.4871	1.538	1	0.2156	1.829	0.7046	4.746
Tumor state	0.4646	0.4862	0.9120	1	0.3400	1.591	0.6131	4.127
First course of radiotherapy	1.411	0.5082	7.733	1	**0.0062**	4.099	1.513	11.10

**Table 6:** COX multifactor analysis.

Subject	*B*	SE	Wald	Df	*P*	Exp (B)	95.00% CI	
							Upper	Lower
MCTC	1.673	0.7799	4.600	1	**0.0319**	5.327	1.155	24.57
TNM stage	1.094	0.4583	5.696	1	**0.0169**	2.986	1.216	7.332
First course of radiotherapy	0.675	0.5185	1.693	1	0.1932	1.963	0.7110	5.425

## Data Availability

The data used to support the findings of this study are available from the corresponding author upon request.
